# MRI Enhancement in Stromal Tissue Surrounding Breast Tumors: Association with Recurrence Free Survival following Neoadjuvant Chemotherapy

**DOI:** 10.1371/journal.pone.0061969

**Published:** 2013-05-07

**Authors:** Ella F. Jones, Sumedha P. Sinha, David C. Newitt, Catherine Klifa, John Kornak, Catherine C. Park, Nola M. Hylton

**Affiliations:** 1 Radiology and Biomedical Imaging, University of California San Francisco, San Francisco, California, United States of America; 2 Epidemiology and Biostatistics, University of California San Francisco, San Francisco, California, United States of America; 3 Radiation Oncology, University of California San Francisco, San Francisco, California, United States of America; King Faisal Specialist Hospital & Research Center, Saudi Arabia

## Abstract

**Rationale and Objectives:**

Normal-appearing stromal tissues surrounding breast tumors can harbor abnormalities that lead to increased risk of local recurrence. The objective of this study was to develop a new imaging methodology to characterize the signal patterns of stromal tissue and to investigate their association with recurrence-free survival following neoadjuvant chemotherapy.

**Materials and Methods:**

Fifty patients with locally-advanced breast cancer were imaged with dynamic contrast-enhanced magnetic resonance imaging (DCE-MRI) before (V1) and after one cycle (V2) of adriamycin-cytoxan therapy. Contrast enhancement in normal-appearing stroma around the tumor was characterized by the mean percent enhancement (PE) and mean signal enhancement ratio (SER) in distance bands of 5 mm from the tumor edge. Global PE and SER were calculated by averaging all stromal bands 5 to 40 mm from tumor. Proximity-dependent PE and SER were analyzed using a linear mixed effects model and Cox proportional hazards model for recurrence-free survival.

**Results:**

The mixed effects model displayed a decreasing radial trend in PE at both V1 and V2. An increasing trend was less pronounced in SER. Survival analysis showed that the hazard ratio estimates for each unit decrease in global SER was statistically significant at V1 [estimated hazard ratio = 0.058, 95% Wald CI (0.003, 1.01), likelihood ratio p = 0.03]; but was not so for V2.

**Conclusions:**

These findings show that stromal tissue outside the tumor can be quantitatively characterized by DCE-MRI, and suggest that stromal enhancement measurements may be further developed for use as a potential predictor of recurrence/disease-free survival following therapy.

## Introduction

The evolution of breast cancer requires co-optation of the surrounding stromal tissues to facilitate progression and support metabolic demand. This has been shown in the earliest stages of breast cancer, ductal carcinoma in situ (DCIS), where proliferating malignant cells inside the breast duct are associated with a remodeled stroma outside of the duct, characterized in part by increased angiogenesis and microvessel density [1]. In addition to increased angiogenesis, there are striking changes in the cellular constituents of the activated cancerous stroma including immune cell infiltrates, [2] remodeling of extracellular matrix [3] and physiologic changes in pH and oxygen tension [4], reflecting increased metabolic demand. The past several years have witnessed a substantial increase in the understanding of the molecular and functional basis of these constituents, creating new opportunities for targeted therapies, and new prognosticators [5]. The challenge of finding new prognosticators among thousands of possible stromal molecular candidates is indeed daunting, and array-based profiling has provided the ability to create signatures to understand a series of molecular changes in aggregate [6].

To translate biologic features of activated stroma that may provide information to enhance individualized patient care, image-based prognostic tools are needed. These tools provide non-invasive access to biologic information, which have obvious merits. Since magnetic resonance imaging (MRI) is being used to diagnostically evaluate breast tumor stage and aggressiveness, and provides an unprecedented ability to evaluate breast anatomy in three dimensions, we questioned whether MRI could be used to further evaluate non-cancerous breast tissue beyond the region of the identified tumor. Gadolinium extravasation forms the basis for MRI-detected enhancement in tumors, and thus the quantitated levels of signal enhancement ratios (SER’s) in the stroma were analyzed. The previously reported evaluation [7] used manually drawn regions of interest to show that global enhancement characteristics in the stroma (based on SER values) had predictive association with higher 3-year disease-free survival (82.4%) in breast cancer patients that underwent neoadjuvant chemotherapy [7]. Subsequent development of a segmentation technique has allowed automatic quantification of stromal enhancement measures, voxel-by-voxel relative to the distance to the tumor [8]. When applied, this tool demonstrated that the peak stromal enhancement was elevated in regions surrounding invasive breast tumors and was associated with increased microvessel density [9]. In the present study, we applied our more advanced tissue mapping technique to validate the previous findings and to determine whether MR enhancement in the stromal tissue could be a predictor for recurrence-free survival following neoadjuvant chemotherapy.

## Materials and Methods

### Ethics Statement

68 patients with stage II or III locally advanced breast cancer were enrolled in a neoadjuvant chemotherapy breast cancer protocol that was reviewed by the UCSF institutional review board and approved by the Committee of Human Research under the UCSF Human Research Protection Program between 1995 and 2002. All patients had given their written informed consent to participate this study.

### Study Population

In this retrospective study, all patients had confirmed diagnosis based on histopathology of biopsy or surgical excision of lesion, and none of them had prior treatment with chemotherapy, surgery or radiation. Five patients were excluded from the analysis for the following reasons: 1) missing pre-treatment MRI; 2) incomplete therapy; 3) deviation from the therapeutic protocol and 4) missing follow-up visit. All 63 remaining patients received four cycles of adriamycin-cytoxan neoadjuvant chemotherapy administered every 3 weeks, and 16 received additional weekly treatment with taxane. These patients were previously reported in a study to assess tumor response to treatment. In this study, we focused on the stromal analysis surrounding the breast tumor.

**Figure 1:**
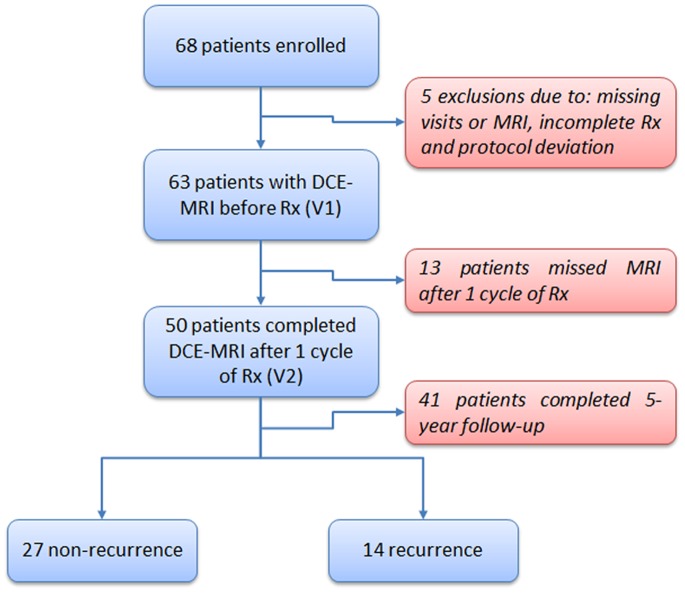
Patient inclusion/exclusion flow chart.

All patients underwent dynamic contrast-enhanced (DCE) MRI before chemotherapy (V1), and 50 patients were scanned after one cycle of chemotherapy (V2) (Figure 1). Recurrence-free survival (RFS) was assessed for each patient based on the absence of palpable mass by clinical examination and suspicious lesion by mammographic imaging (at 6-month or 1-year intervals) following surgery and recurrence categorized as local or distant. The length of RFS was defined as the time from initial surgery to either local or distant recurrence or the time to the last follow-up in patients without evidence of recurrence. Lesion characteristics such as pretreatment tumor size measured by volume, tumor longest diameter and pathology, nodal involvement and Scharff-Bloom-Richardson (SBR) grading were recorded.

### MRI Acquisition

MRI was performed on the tumor bearing breast (ipsilateral) only. Images were acquired on a 1.5-T scanner (Signa, GE Healthcare, Milwaukee, WI) using a dedicated bilateral phased-array breast coil. A fat suppressed T1-weighted 3-D fast gradient-recalled echo sequence was used (TR/TE, 8/4.2; flip angle, 20°; 2 repetitions) [10]. Gadopentetate dimeglumine (Magnevist, Bayer HealthCare) was used as a contrast agent, and was injected at a dose of 0.1 mmol/kg of body weight (2 mL per second) followed by a 10 mL saline flush. The entire breast was scanned sagittally with 60 slices of 2 mm thickness and a total scanning time of approximately 5 minutes [11]. Low order phase-encoding data were acquired at the center of the scan, resulting in an effective time point of 2.5 min from the start of the scan. Three time points were acquired during each MRI examination: a baseline scan before contrast agent injection (*t_0_*), followed by 2 time points measured in the early (*t_1_*) and late phases (*t_2_*) after contrast injection, yielding temporal post-contrast sampling times of approximately 2.5 and 7.5 minutes, respectively [11]. Despite the long scan duration, the first post-contrast time sample occurred at 2.5 min using the standard k-space sampling, which was close to the effective sampling of 3 min or less that was recommended by the American College of Radiology guideline for breast MRI [12].

### Percent Enhancement (PE) and Signal Enhancement Ratio (SER) Analysis

**Figure 2:**
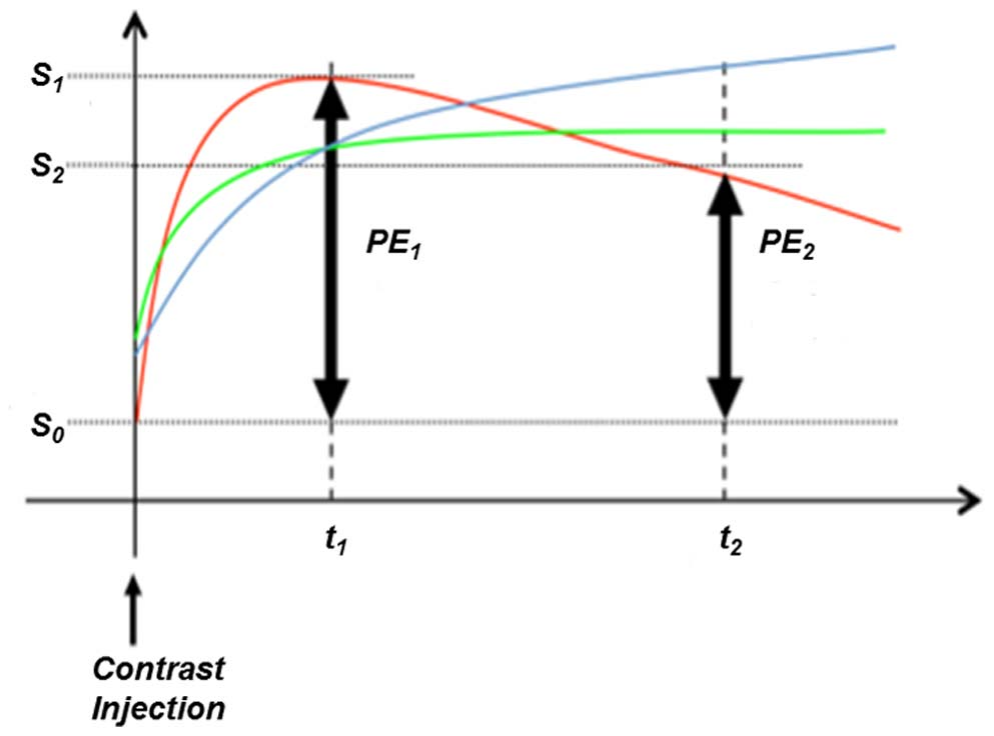
Signal intensity-time curve for DCE-MRI. Signal intensity-time curve for DCE-MRI showing early (PE*_1_*) and late (PE*_2_*) percent enhancement measurements. S*_0_*, S*_1_*, and S*_2_* represent the signal intensity of images obtained at *t_0_* (before contrast injection), *t_1_* (2.5 minutes after contrast injection), and *t_2_* (7.5 minutes after contrast injection), respectively. Three curves display different patterns of signal increase and washout: 1) the blue curve shows a slow gradual increase in enhancement, more characteristic of normal tissue; 2) the green curve shows an early enhancement with little washout, essentially a plateau in signal intensity; 3) the red curve shows a pattern of early enhancement with a fast washout, which is more characteristic of highly vascularized tissues.

In breast DCE-MRI, physiological parameters related to tumor vascularity can be extracted from the contrast enhancement kinetics exhibited in the signal intensity-time curves (Figure 2). Malignant tissues are characterized by a rapid rise in signal intensity after contrast injection followed by a stabilized signal intensity or signal washout (Figure 2, red curve). Normal and benign tissues, by comparison, show a slower increase in enhancement with little or no washout (Figure 2, blue and green curves). In the signal intensity-time curve, S*_0_*, S*_1_* and S*_2_* are the signal intensity values in the pre-contrast (*t_0_*), early post-contrast (*t_1_*) and late post-contrast phases (*t_2_*), respectively (Figure 2). Percent enhancement (PE = 100*(S*_1_*-S*_0_*)/S*_0_*) is calculated for each voxel. Signal enhancement ratio (SER), defined as the ratio of early to late enhancement (SER = (S*_1_*-S*_0_*)/(S*_2_*-S*_0_*)), is a method developed to measure contrast enhancement kinetics from high spatial resolution, low temporal resolution DCE-MR images commonly used for clinical breast MRI [13]. High SER values consistently identify tissue with a strong signal washout characteristic [14].

### Proximity Mapping of Breast Stroma

**Figure 3:**
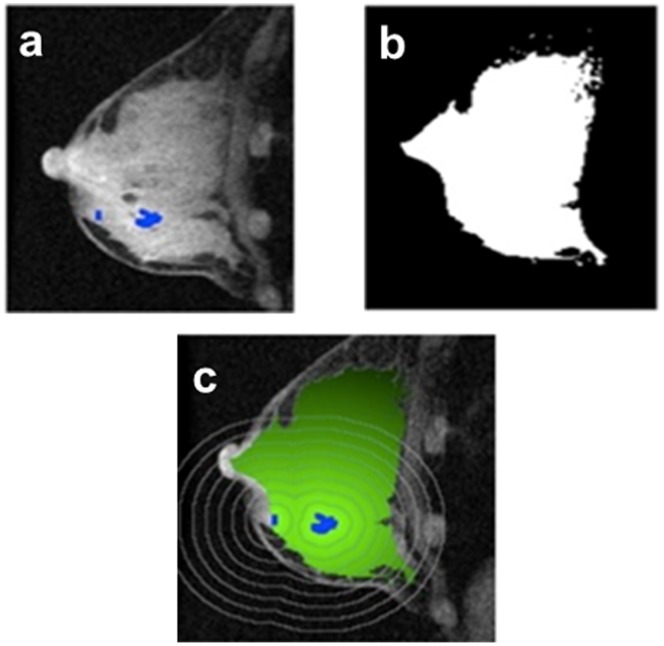
Proximity mapping of breast tumor. The proximity mapping method takes the difference of the segmentation of tumor (a) and fibroglandular map (b) to create a 3-dimensional stromal proximity map (c). The stromal proximity map is then applied to the functional image to calculate proximity-dependent values.

Tumor regions on MR images were identified using an established enhancement criteria of 70% applied to the first post-contrast image [15]. This empirical threshold was based on visual agreement with radiological assessments in clinical practice [11]. Normal-appearing stromal tissue surrounding the tumor was subsequently defined as fibroglandular tissue and was segmented from adipose tissue using a fuzzy C-means clustering method [8]. Subsequent PE and SER maps were generated using a customized software program that was previously described [15]. The tumor proximity map for normal-appearing breast stroma was generated by calculating the 3-D map of the Euclidean distance between each non-tumor voxel and the nearest tumor voxel. The proximity map was then overlaid using the segmented fibroglandular tissue mask and applied to the PE and SER maps to calculate the mean PE and SER values in 3-D distance bands of 5 mm, from 0 to 40 mm outside of tumor tissue (Figure 3). Global PE and SER values were the average of all the mean PE and SER values over 5 to 40 mm. Region closest to the tumor boundary from 0 to 5 mm was considered as tumor periphery [16]. All subsequent calculations of stromal effects on radial distance, global PE and SER were focused at regions from 5 to 40 mm.

### Statistical Analysis

The purpose of this study was to evaluate the stromal enhancement patterns and to determine whether proximity-dependent enhancement was associated with RFS following neoadjuvant chemotherapy. Mixed effects modeling with a linear relationship between the predictor radial distance and outcomes of MR stromal enhancement (with subject-specific random intercepts and slopes) was used to evaluate the radial trend of PE and SER in the range of 5 to 40 mm from the tumor border. When considering RFS as a predictor rather than outcome, RFS was defined based on status (recurrent vs. non-recurrent) specifically at 5-years in order to ensure against bias due to differential follow-up times. The 5-year cut-off led to 21 patients defined as recurrent and 32 patients as non-recurrent at 5 years. The Wilcoxon-Mann-Whitney U-test (rank-sum) was used to analyze the difference of PE and SER values in the recurrent and non-recurrent 5-year status (two-sided tests were used) and results were reported with estimated (pseudo-) median differences and 95% confidence intervals. Single predictor Cox proportional hazards modeling was used for survival analysis with time to events of RFS as the response variable and global PE/SER measurements as the predictors. (The Cox model explicitly accounts for differential follow up so no cut-point for defining recurrence was required for this analysis.) For the analysis of MR stromal enhancement, PE and SER obtained from MRI at V1 and V2 were considered. All Cox proportional hazards results were reported as estimated hazard ratios, Wald 95% confidence intervals and likelihood ratio test p-values. All statistical analyses were performed using the R statistical analysis software package. A nominal statistical significance level of α = 0.05 was used throughout.

## Results

### Patient Characteristics

Patient age ranged from 30 to 72 with the median at 48 and inter-quartile range of 14. There were 38 (<50, 60.3%) pre-menopausal and 25 (≥50, 39.7%) post-menopausal patients. The Cox proportional hazards analysis showed that age was not a statistically significant predictor of RFS (mean = 48; Hazard ratio (HR) = 0.974, 95% CI (0.935, 1.027), p = 0.2). When the cohort was divided into 5-year non-recurrent and recurrent groups, there were 32 non-recurrent cases, of which 22 completed both MR scans at V1 and V2. The recurrent group comprised 21 patients and 14 completed both MR scans at V1 and V2.

### Tumor Characteristics in Association with Recurrence-Free Survival

In this cohort of 63 patients, the Cox proportional hazards analysis showed that pre-treatment tumor size measured by volume (median = 16.5 cm^3^; Hazard ratio (HR) = 1.017, 95% CI (1.006, 1.027), p = 0.002, i.e., there was a 1.7% increase in instantaneous risk (or hazard) for each unit increase in volume), longest diameter (median = 5.12 cm; HR = 1.220, 95% CI (1.059, 1.405), p = 0.006) and pathological size at resection (median = 2.50 cm; HR = 1.164, 95% CI (1.018, 1.331), p = 0.03) had statistically significant association with RFS. In addition, the number of axillary nodes involved (median = 1; HR = 1.074, 95% CI (1.010, 1.141), p = 0.03) was also a statistically significant predictor. Prediction of RFS by other characteristics including pathological subtype and SBR grading were not statistically significant: details of the estimated relationships between these patient tumor characteristics and RFS in terms of hazard ratios, confidence intervals and p-values can be found in Table 1.

**Table 1:** Patient and Tumor Characteristics.

Characteristics	Value	Hazard Ratio per unit change (95% CI)	P
**Patient Age**		0.974 (0.935–1.027)	0.2
<50	38		
> = 50	25		
**Pretreatment Tumor Size**			
Longest diameter (median, interquartile range in cm)	5.12 (3.44–7.66)	**1.220 (1.059–1.405)**	**0.006**
Volume measured by MRI (median, interquartile range in cm^3^)	16.5 (5.77–43.1)	**1.017 (1.006–1.027)**	**0.002**
Pathologic tumor size at resection (median, interquartilerange in cm) (n = 62, 1 missing)	2.50 (1.30–5.33)	**1.164 (1.018–1.331)**	**0.03**
**Nodal Status (n = 62)**			
No. positive nodes (median, interquartile rage)	1 (0–4)	**1.074 (1.010–1.141)**	**0.03**
Patients with positive nodal involvement	40		
Patients without positive nodal involvement	22		
Pathology Class (analyzed as continuous)*			
No. Patients		1.387 (0.856–2.246)	0.2
Class 1 = No residual disease or pCR	7		
Class 2 = <1 cm residual disease	5		
Class 3 = 1 cm <residual disease <2.5 cm	19		
Class 4 = >2.5 cm residual disease	30		
Scharff-Bloom-Richardson grade (n = 51) (analyzed as continuous)*		1.206 (0.912–1.597)	0.2
No. Patients			
1	13		
2	21		
3	17		

### Stroma Enhancement Patterns

Low-level contrast enhancement patterns in normal appearing stroma surrounding breast tumors were evaluated using DCE-MRI. Mean PE and SER values at 5 mm increments were plotted against the radial distance 5–40 mm from the tumor edge.

#### Percent enhancement pattern

For all patients, PE values continuously decreased at both V1 and V2 from the tumor proximal tissue (5–10 mm) to distal stromal tissue (35–40 mm). The linear mixed effects model, including stromal distance from 5 to 40 mm, displayed this decreasing radial trend at V1 (estimated mean change per mm = −0.399, 95% CI (−0.501, −0.296), p<0.0001) and V2 (−0.343, 95% CI (−0.557, −0.128), p = 0.002).

**Figure 4:**
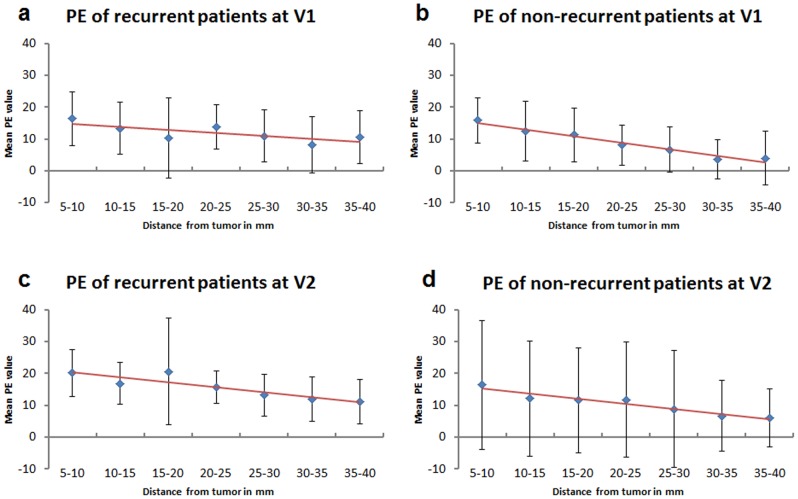
Radial trend of PE in recurrent and non-recurrent patients. Radial trends of PE values in recurrent and non-recurrent groups (based on a 5-year cut off) were shown here over a distance of 5 to 40 mm from the tumor edge. A decreasing trend (shown in red trend line) of PE was observed in all cases (3a–d).

When the recurrence status was added as a group variable to the mixed effects model, PE exhibited the same decreasing radial trend in both recurrent and non-recurrent groups at V1 and V2 (Figure 4). At V1, the estimated mean PE change per mm in the non-recurrent group was -0.416, 95% CI (−0.572, −0.259), p<0.0001 and in the recurrent group was −0.453, 95% CI (−0.648, −0.257), p<0.0001. The estimated difference in distance effects between the two groups (recurrent minus non-recurrent) was negative but small (the recurrence group having greater decline with distance) and with a wide confidence interval (−0.037, 95% CI (−0.288, 0.213), p = 0.8). At V2, the estimated mean PE change per mm in the non-recurrent group was −0.502, 95% CI (−0.809, −0.195), p = 0.002, but in the recurrent group was estimated as −0.148, 95% CI (−0.564, 0.268), p <0.5. The estimated difference between the slopes (with respect to radial distance) of the two groups was positive, but with a wide confidence interval (0.354, 95% CI (−0.163, 0.872), p = 0.2).

We also investigated the change in PE between V1 and V2 at the tumor periphery (0–5 mm). In the non-recurrent group, the PE values were estimated to be lower (approaching statistically significantly) at V2 compared to V1 (estimated (pseudo-) median difference = 3.91, 95% CI (−0.36, 8.73), two-tailed Wilcoxon signed rank test, p = 0.07). However, the difference in the recurrent group was not statistically significant (estimated median difference = 0.210, 95% CI (−2.05, 4.18), p = 0.7).

#### Signal enhancement ratio pattern

The radial trend was less pronounced in SER over the same distance range at either V1 or V2 and did not achieve statistical significance: at V1 there was an estimated mean increase in SER of 0.0022 per mm, 95% CI (−0.0006, 0.0049), p = 0.2, with a similar estimate at V2, 0.0022 per mm, 95% CI (−0.0005, 0.0050), p = 0.2.

**Figure 5:**
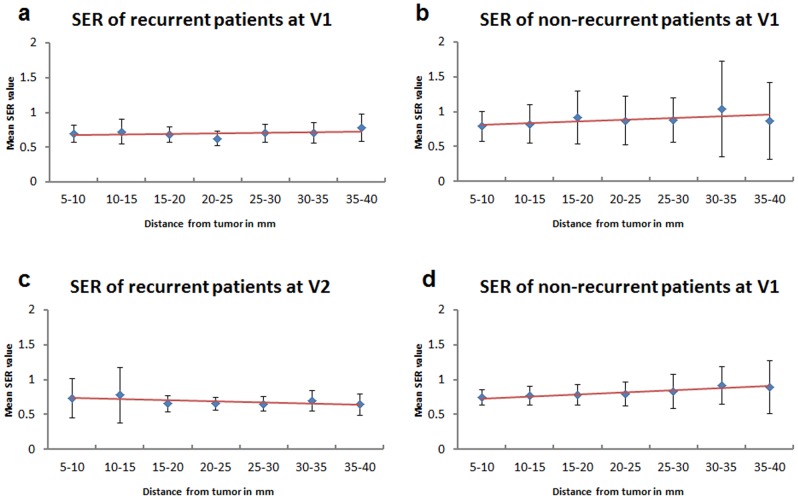
Stromal enhancement pattern of SER in recurrent and non-recurrent patients. Stromal enhancement pattern of SER in recurrent and non-recurrent cohorts at V1 and V2 (based on a 5-year cut-off). The red line shows the trending of SER. At V1: there was no observable trend for recurrent group (a) but an increasing trend of stromal SER outside of tumor was observed in the non-current group (b). At V2: No observable trend was found in the recurrent group (c), but a slight increasing trend was found in the non-recurrent group (d).

When the recurrence status was added as a group variable to the mixed effects model, SER showed a slight (but not statistically significant) increasing trend in both non-recurrent (V1: 0.0025, 95% CI (−0.0017, 0.0067), p = 0.3; V2: 0.0023, 95% CI (−0.0018, 0.0064), p = 0.3) and recurrent groups (V1: 0.0009, 95% CI (−0.0046, 0.0064), p = 0.8; V2: 0.0031, 95% CI (−0.0026, 0.0089), p = 0.3) (Figure 5). There was no evidence of a difference in pattern in non-recurrent versus recurrent groups with estimated differences (recurrent minus non-recurrent) in slopes close to zero, though the confidence intervals were wide: (V1: −0.0016, 95% CI (−0.0085, 0.0053), p = 0.7; V2: 0.0008, 95% CI (−0.0063, 0.0078), p = 0.9). Finally, when examining difference in distance effects related to recurrent and non-recurrent groups, there were no statistically significant results to report and confidence intervals were too wide to make any conclusions with respect to negative results.

One additional note is that we did observe mild departures from normality in the residuals of the linear mixed-effects models in normal QQ-plots. Therefore, there may be some bias in our model results. This potential non-normality should be examined further in similar analysis of future datasets.

### Global PE and SER in Recurrent and Non-recurrent Patients

For global PE at V1, there was no clear difference found between recurrent and non-recurrent patients (estimated (pseudo-) median difference (recurrent minus non-recurrent) = 1.06, 95% CI (−3.09, 5.31), two-tail Wilcoxon-Mann-Whitney U-test p = 0.6), but there was a statistically significant difference at V2 (8.24, 95% CI (2.93, 12.27), p = 0.02). A statistically significant lower global SER was found in the recurrent group compared to non-recurrent group at V1 (estimated median difference = −0.081, 95% CI (−0.157, −0.002), p = 0.04) but not at V2 (−0.058, 95% CI (−0.152, 0.015), p = 0.3).

### Recurrence-free Survival Analysis

Survival analysis using the Cox proportional hazards model with global PE or SER as predictors showed that for each unit increase in PE there was an estimated hazard ratio of 1.02, Wald 95% CI (0.97, 1.07), likelihood ratio p = 0.5 for V1; and 1.02, 95% CI (0.994, 1.05), p = 0.2 for V2 indicating no clear effect of global PE on risk of recurrence. Whereas, for each unit decrease in SER the estimated hazard ratio of 0.058, 95% CI (0.003, 1.01), p = 0.03 for V1 was statistically significant (note that the p-value is based on the likelihood ratio test, whereas the confidence interval is the Wald confidence interval, hence the apparent discrepancy between p<0.05 and the confidence interval crossing 1.0); but was not so for V2: estimate = 0.644, 95% CI (0.0253, 16.4), p = 0.8, indicating a possible relationship between SER at V1 and risk of recurrence. Tests of proportional hazards for all models did not indicate significant departures from the proportional hazards assumption with p-values: 0.1 (PE V1), 0.4 (PE V2), 0.6 (SER V1), and 0.3 (SER V2).

## Discussion

In the past decade, research in cancer biology has provided new insights into the tumor-promoting functions of stroma in breast cancer. In the presence of tumor cells, stroma produces critical signals to drive proliferation, angiogenesis and motility while suppressing apoptosis [3]. In the absence of pre-existing tumor cells, stroma can also acquire genomic changes to stimulate the transformation of adjacent cells and to ultimately facilitate malignancy [17]. These indicators of activated stroma may give rise to characteristic morphological features such as change in cellularity and/or vascularity that may be assessed by image-based tools. Therefore, it is important to develop new imaging methodology to assess stromal tissues before the manifestation of tumor growth and relapse. In fact, mammographically detected breast density [18], [19] and high water content measured by MRI [20] are thought to reflect increased cellularity from proliferation of epithelium and stroma. Moreover, dynamic techniques using contrast-enhanced MRI further provide quantitative parameters such as peak contrast-enhancement, k*^trans^* [21] and signal enhancement ratio (SER), that are correlated to intratumoral microvessel density and vascular endothelial growth factor (VEGF) expression [22]. We reasoned that these imaging parameters can be used to characterize the extratumoral morphologic features of stroma and may convey information about clinical outcome.

In a previous study, SER analysis of normal-appearing breast stroma was manually performed by defining and placing regions of interest (ROI) radially from the tumor edge [7]. In the current study, the semi-automated iterative segmentation technique and tumor proximity map has enabled us to calculate stromal enhancement values more precisely at specific distances around the tumor. This methodology can be applied to any registered functional image, such as PE, SER, apparent diffusion coefficient (ADC) or fractional anisotropy (FA), making it a versatile and robust method for stromal characterization.

In this cohort of 63 patients, the univariate Cox analysis showed a statistically significant association with RFS for initial tumor volume and diameter measured by MRI. A 1.7% increase in hazard of recurrence was found for each unit volume increase. Although 1.7% increase appears to be small, it can be clinically significant when a unit change in the predictor is small compared to the spread in the population; the hazard of recurrence increases exponentially (is compounded) with each additional unit change in the predictor and therefore a realistic 10 cm^3^ difference in tumor size between two patients would have a clinically important effect on their relative hazard (18% increase for the larger tumor size). This interpretation agrees with that of Gray [23] who determined a low hazard ratio of longest diameter for predicting survival, but which was nevertheless clinically important for meaningful diameter differences.

In assessing the tumor periphery region, signal enhancement was dominated by the permeability of the gadolinium contrast agent through capillary walls in angiogenic vasculature [16] measured by PE. Previously, it has shown that an increased PE in the tumor periphery was associated with increased microvessel density [9]. Based on these findings, we reasoned that this measurement may be also responsive to chemotherapy. In the non-recurrent group, PE values immediately outside of the tumor (0–5 mm) after an initial exposure to chemotherapy (at V2) were estimated lower with a reduction of 3.91, 95% CI (−0.36, 8.73), p = 0.07) than that at V1, possibly reflecting the inhibitory effect of chemotherapy at the tumor periphery angiogenesis.

While PE measures signal enhancement through vascular permeability of the contrast agent, SER assesses the contrast washout kinetics from the tissue. In this study, although the increasing radial trend in SER was not statistically significant, a coherent pattern of increasing estimated SER can be visualized in the non-recurrent cohort at V2 (red trend line in Figure 5d). This trend was in agreement with previous findings that higher global stromal SER values (> 0.7) after one cycle of chemotherapy (V2) were associated with reduced risk of recurrence [7].

To characterize an overall stroma enhancement, the global PE and SER values averaged over the range of 5 to 40 mm was analyzed. A statistically significant difference between recurrent and non-recurrent groups was found in global PE at V2 (8.24, 95% CI (2.93, 12.27), p = 0.02) and global SER at V1 (−0.081, 95% CI (−0.157, −0.002), p = 0.04), indicating that these global measurements may reflect the overall difference of tissue biology in recurrent and non-recurrent patients and their response to therapy. In the Cox proportional hazards model for RFS, global SER at V1 was found to be a statistically significant predictor of RFS (p = 0.03): for every unit decrease in SER, the estimated hazard was increased by a factor of over 17. Although the confidence interval was very wide, this finding further supports the observation of increasing radial trend of stromal SER values in non-recurrent patients, as well as previous findings of higher stromal SER in association with reduced risk of recurrence. The higher stromal SER may reflect greater microvessel density to facilitate delivery of chemotherapeutic agents, resulting in greater efficacy and reduced risk of recurrence.

The current study population is limited to women with advanced stage disease who received neoadjuvant chemotherapy. Another limitation is that there was only a small subset of the recurrent group with both MRI scans at V1 and V2. These limitations impact our ability to generalize our findings to patients with better prognosis. We recognized that our survival analyses may be influenced by the size of the measurable stromal tissue. To address this, we performed a sensitivity analysis via Cox proportional hazard modeling by incorporating stromal size as an additional covariate in each of the survival models for global PE and SER at V1 and V2. Stromal size was not a statistically significant covariate in any of the models and moreover, did not affect the general relationship between SER at V1 and recurrence. While these results do not prove that stromal size has no predictive value, they indicate that the predictive power of stromal size is limited and in particular does not appear to diminish the predictive effect of SER at V1. The current segmentation technique may pose an issue of partial volume that can be addressed by implementing an erosion correction in the future. The current study has demonstrated a new robust image-based tool for stromal characterization. Our continued efforts to refine proximity analysis of PE and SER, as well as extending the proximity mapping methodology to study the contralateral breast with additional descriptors such as ADC [24] or FA [25] measurements may provide additional predictive stromal imaging characteristics that can be further developed for individualized treatment intervention.
